# Participants' Perspectives of a Primary Exercise-Based Prevention Program for Cardiac Patients: A Prepost Intervention Qualitative Case Study

**DOI:** 10.1155/2020/6215428

**Published:** 2020-04-15

**Authors:** Mélissa Lesage-Moussavou-Nzamba, Julie Houle, François Trudeau

**Affiliations:** ^1^Department of Human Kinetics, Université du Québec à Trois-Rivières, Trois-Rivières, Québec, Canada; ^2^School of Nursing Sciences, Université du Québec à Trois-Rivières, Trois-Rivières, Québec, Canada

## Abstract

Perseverance in exercise-based, cardiovascular disease prevention programs is generally very low. The purpose of this case study is to understand the experience of participants enrolled in a 6-month primary and secondary exercise-focused, cardiovascular disease prevention out of hospital program. Ten participants were interviewed about their experiences at entry and after it ended 6 months later to understand the facilitators and difficulties encountered by participants in such exercise programs. Four out of ten participants completed the 6-month program. The six participants who left the program accepted to contribute to the postprogram interview. The results showed that the four participants who persevered in the program became aware of cardiac risk factors and their conditions were willing to make changes in their lifestyles to reach their objectives, felt a strong perception of self-efficacy, and felt like they belonged in the program. Both persevering and nonpersevering participants experienced many episodes of discouragement during the program and faced many barriers that interfered with their progress. Suggestions to help coping with these barriers while reinforcing self-efficacy and the sentiment of belonging are discussed.

## 1. Introduction and Statement of Purpose

Cardiovascular diseases are significantly prevalent worldwide and are one of the two major causes of mortality [1]. As many as 1.3 million Canadians were impacted by cardiovascular disease in 2007, and a great majority of them (68.8%) reported limitations of leisure activities [2]. Ischemic cardiopathy, the most common form of cardiovascular disease, regroups angina and myocardial infarction [3]. Its prevalence impacts 35% of the Québec population aged 70 years or older [4].

Participation in exercise-based, cardiac-rehabilitation programs and the adoption of healthy life habits are known to significantly diminish the negative consequences of cardiovascular disease [5]. The efficacy and cost-benefit ratio of exercise programs for primary and secondary prevention in people with heart diseases or those with risk factors for the development of these diseases are well documented in the literature and show that cardiac rehabilitation is cost-effective [6, 7, 8]. Regular physical activity provides many health benefits to these patients such as reduced hospital readmission [9], ability to perform activities of daily living, quality of life, risk factor profile, and exercise capacity [6]. However, these programs are not widespread. Where they exist, few patients are referred to them and, once admitted, fewer still persist with them [10].

Given such low participation and high dropout rates in these programs, it is important to understand the experiences of cardiovascular disease patients. Many researchers have examined the determinants of participation and adherence to cardiac rehabilitation programs. These determinants are a perception of self-efficacy, self-motivation, self-confidence, personality traits, depression, anxiety, support, multiple responsibilities, time, and finances [11, 12, 13]. Adherence to, participation, and attendance in such programs for cardiac rehabilitation have been mainly studied through quantitative approaches and qualitative studies not included in reviews [14, 15]. Few researchers have examined the phenomenon of persistence from the perspective of experience in primary and secondary exercise-based prevention programs before and after such an intervention.

The purpose of our case study was to better understand the experience of participants in a physical activity program aimed at secondary cardiovascular disease prevention. This qualitative approach was designed to understand the experience of participants in a primary and secondary exercise-based, cardiovascular disease prevention program, in those who completed the 6-month program, as well as those who dropped out. The main research question was to understand barriers and facilitators that were perceived in participants that either persevere or dropout of an out-of-hospital program.

## 2. Methods

### 2.1. Case Study Design

The choice of this intervention as the object of this case study is linked to the fact that most patients do not have access to medically supervised cardiac or metabolic rehabilitation for the rest of their life following a diagnosis of cardiometabolic problems. In fact, most of the patients are without sufficient risks to warrant a tightly supervised exercise program for prevention (either primary or secondary). Therefore, out-of-hospital exercise centers with kinesiologists (in other countries/states: exercise specialists of exercise physiologists) may be the most prevalent and accessible venue for exercising in this population.

### 2.2. Case Study Participants

The current study was approved by the Université du Québec à Trois-Rivières Research Ethics Committee. Sampling was performed through convenience sampling. A kinesiology clinic was approached to solicit their clients for volunteering into participating in an exercise-focused primary and secondary cardiovascular disease prevention program. The clinic was located in a metropolitan area of Montréal (Québec, Canada). The criterion for inclusion in the study was (1) having one or more cardiovascular disease risk factors and (2) being medically cleared to participate. No other selection criteria were used, and all interested participants who volunteered were included in the study. At the first interview, patients were asked if they were interested in participating freely in a 6-month case study with all information being kept confidential and anonymous. Duties of the participants were exposed during this meeting. They were told to determine an involvement contract and sign it. The contract was revised periodically and new objectives were settled. Each client had to communicate with their kinesiologist about any health problem or other problems that may be related to their training. They commit themselves to notify the clinic if they were unable to attend their training and to mention the reason for the absence, if possible. Participants were advised that they retained the right to leave the program at any time. The characteristics of the study participants are listed in Table 1. Of the participants, 2 were women and 8 were men. The mean age was 51 ± 11 years, ranging from 35 to 75 years. The study sample was largely composed of men with a homogenous ethnic background: 7 males and 2 females of European descent and 1 Hispanic male. One participant (P5) abandoned the program and did not return our calls to participate in the second interview. We therefore did not include this participant in our analysis.

### 2.3. Context of the Program/Intervention

The program is aimed at helping participants change their lifestyle habits through physical activity, incorporating education about cardiovascular disease risk factors, and coaching with regular follow-ups. In addition to individual training (aerobic activities, muscular conditioning, proprioception, and flexibility), participants were given pamphlets on healthy eating, physical activity, and heart disease-related risk factors. The frequency, duration, and modality of training varied across the program according to agreements between participants and kinesiologists. Participants were strongly encouraged to record their activities, state of health, absences, and progress in a logbook. Under supervision by kinesiologists, they also had to choose a realistic short-term goal that they wanted to accomplish and write it down in a contract of commitment to the program. Each participant had to keep in mind only one objective at a time, and a deadline was set for its completion. When the goal was reached and maintained, the kinesiologists had to follow-up with participants to review the target, set a second target, and so on. The kinesiologists were expected to help participants define a specific plan of action to accompany each goal, as in this example:

“I want to feel less breathless when I go up the stairs by the end of June. I will try to accomplish my goal by introducing 30 minutes of cardiovascular activities in my daily life at least 5 times a week.”

### 2.4. Protocol of Questionnaires and Interviews

Each participant completed an entry questionnaire, including information on sociodemographic characteristics and medical history (e.g., age, marital status, occupation, risk factors, and health problems) as well as desired objectives. Entry and exit interviews of each participant enabled us to learn about his/her perceptions of cardiovascular disease prevention programs and the interaction of these perceptions with the main program components. Even participants who dropped out before the end of the program were interviewed postintervention. The primary objective of the interviews was to identify factors that influence participation in and adherence to the programs. The starting interview questions were, for example:

“Tell me about the experiences you have had in trying to change your lifestyle in your daily life or within the program.”

Participants were encouraged to openly discuss, for example, their fears, what could motivate or disable them during the program, their goals, previous experiences with lifestyle changes and coaching, or any other factors that could potentially influence progression toward their goals. Participants who dropped out were recontacted for a second interview to ascertain why they had dropped out and their views on the program.

The concluding interview, conducted 6 months after the first one, dealt substantially with the same participants to identify differences in perceptions, emotions, and experiences after participating in the program. No minimum or maximum time was allotted for interviews, which were conducted in a separate office of the Kinesiology Clinic with the interviewer asking questions and recording the statements.

### 2.5. Data Collection and Qualitative Analysis

All interviews were captured using an Olympus DS50 recorder to ensure accuracy and relevance. Recordings were transcribed verbatim. The first author read all transcripts the first time and coded the data during each subsequent reading. After the first author finished a first theme extraction, she presented the themes and subthemes to the last author for intercoding. When disagreements occurred during intercoding, a third researcher was asked to intervene in the discussion. Analysis of the 2 verbatim interviews made it possible to compare them and to see the relationship between participation, progress in the cardiovascular disease prevention program, and health. A case study analysis, as conducted in qualitative studies by Yin [16] and Hyett et al. [17], captured the personal experience and perception of events within the program. Qualitative analysis resulted in the formation (coding) of themes and subthemes, including their frequency, to facilitate the understanding of some factors related to participation in and adherence to a primary and secondary cardiovascular disease prevention program (Table 1). Themes and subthemes were compared between the first and second interviews for flexibility in the emergence of themes.

### 2.6. Analysis of Other Program Components

To increase the credibility of findings and interpretations, in addition to interviews, we analyzed all participants' attendance in activities, success rates with their goals and interviews with the kinesiologists who linked their impressions before and after training, and the risk factors identified by them. Perceptions of heart disease prevention were also compared between participants who completed the program and those who dropped out. Adherence to activities was determined by comparing attendance frequency initially suggested by participants with average frequencies at which they were active throughout the program. The number of individual objectives achieved within the program was analyzed using their objectives found in their individual achievement contract. With the factors proposed, we were able to look at positive and negative aspects of the primary and secondary cardiovascular disease prevention program in question and suggest a plan for possible improvement of cardiac rehabilitation.

## 3. Results

### 3.1. Participants' Characteristics

The mean body mass index was 30.20 ± 5.97, ranging from 19.73 to 38.37, and the average fat percentage was 30.42 ± 7.97% with values between 13.7 and 39.9% (Table 1).

#### 3.1.1. Preintervention Questionnaires

Participants self-assessed their risk factors at the first interview. Sedentary lifestyles, excess weight, and diets rich in transfats and sugar were apparent (Table 1). The main reasons they participated in the program-targeted wellness, better health, and weight control (Table 2). The majority of participants attributed their inactivity before the program to a lack of time and interest (Table 3).

At the last interview, 4 (P1, P9, P10, and P11) of the 10 participants were still active at the clinic and continued training on a regular basis. Three (P2, P3, and P8) of the remaining 6 participants had temporarily stopped training at the clinic and at home for health reasons (P2: fatigue and hospitalization due to viral myocarditis; P3: hospitalization secondary to cancer; P8: postdialysis fatigue). Three participants (P4, P6, and P7) decided, for various reasons, to discontinue the cardiac rehabilitation program after their membership subscription expired. All 10 participants, even those who were no longer active with the program, agreed to contribute to the second interview and exit questionnaires 6 months after they entered the programme.

### 3.2. Pre- and Postinterview Analysis

Four main themes emerged from the pre- and postinterviews. They were (1) knowledge and awareness of the disease, (2) perception of personal efficacy, (3) motivation, and (4) feelings of belonging in the program and the relationship with trust. These themes and accompanying subthemes are detailed in Table 4 and discussed below. They were compared between completers and noncompleters in the first and second interviews (Table 5).

## 4. Discussion

Identification codes are explained with the following abbreviations: P# signifies participants (e.g., P1); INT1 and INT2 are the first and second interviews, respectively; O and NO stand for participants who, respectively, completed and did not complete the program.

### 4.1. Theme No. 1: Identifying Alarm Signals: Knowledge and Awareness of the Disease

#### 4.1.1. Risk Factors

Participants' knowledge or awareness of their health condition is one of the factors that may influence the rate of participation in physical activity programs designed to prevent cardiovascular disease.

Participants indicated that understanding the CV risk factors is complex. Through verbatim recordings, participants who did not detect such warning signals before disease onset reported that their vision or source of motivation was less focused on the treatment of their disease (e.g., atherosclerosis, myocardial infarction, and hypertension) than those who sensed them:

“With the infarction, it was the first time in my life I thought that maybe I should stop smoking. It was only after the infarction that I decided to enroll in the program to improve my health.” (P6, INT1, and NO)

“I want to survive, so it's important for me to change some habits.” (P8, INT1, and NO)

Other participants who had several cardiac risk factors joined the program to prevent the development of heart disease. These participants pinpointed several reasons for their involvement in the cardiovascular disease prevention program, mostly realizing that each factor was the cause of the previous one, and that they had to do something to slow the process leading to a cardiac infarction:

“I have borderline hypertension and cholesterol. I need blood tests every month to check my cholesterol level.” (P10, INT1, and O)

“I am concerned because I want to live longer in health. I felt that with age, I was more tired …I had back problems…I had to decide to take charge now if I wanted to be still functional and have a good quality of life in 10-15 years…before it is too late.” (P11, INT1, and O)

Start-up interviews suggested that physicians, friends, and family impacted the awareness that people had about their health and an understanding of these warning signs:

“It started with my doctor...he told me I had high blood pressure.” (P1, INT1, and O)

This is consistent with the literature, which indicates that, although a minority of patients is referred to a cardiac rehabilitation program, a variety of people may influence them to join a program, including their attending physician [18]. Also, when people are aware of cardiovascular disease risk factors and know the source of their health problems, they are more likely to identify and intervene to avoid, treat, or delay the disease [19].

#### 4.1.2. Identification of Causes versus Understanding of the Problem

Interviews revealed that both completers and noncompleters identified risk factors related to their health problem, and the evidence indicated that they acquired new knowledge during the program:

“I eat less fat than before, less sugar, but more fiber…” (P6, INT2, and O)

“I think there is a lot of heredity in the family, arteriosclerosis, cholesterol...which prompted me to undertake a prevention program. Heredity is one of the strongest factors in statistics on the onset of the disease...it is necessary to be more conscious, to avoid harming oneself more.” (P11, INT2, and O)

Although program noncompleters were also aware of their health problems, some were somewhat less willing to make changes:

“I smoke but for me this is not a factor because I don't smoke too much (1 pack per day). In my mind, I will smoke all my life, period.” (P6, INT1, INT2, and NO)

Such a significant positive relationship has been demonstrated between knowledge of cardiovascular disease risk factors and adherence to some lifestyle changes, such as weight control, physical activity, and eating habits [20].

#### 4.1.3. Get to Know Themselves Better and Identify Barriers to and Realities of Physical Activity Practice

Through the program, it seems like participants learned to know themselves better and to identify, for example, what type of individuals they could be in a training context or lifestyle-changing process:

“Training is not something natural to me, so I have to motivate myself more than others.” (P11, INT2, and O)

“…I really need motivation...there are 2 groups of people. I need someone like a kinesiologist to encourage me.” (P3, INT2, and NO)

Barriers were mentioned by all participants. Identifying barriers to physical activity can help kinesiologists (and individuals themselves) to take charge of the situation by finding solutions or ways to adapt. When these individuals have the ability to perceive solutions, preferably early in the program, kinesiologists could work with them so that they remain motivated, and barriers do not prevent them from achieving their goals. In the preparticipation interview, participants spoke, at that time, about lack of motivation, self-discipline, supervision, time, discouragement, stress, and conditions or themes that prevented them from practicing physical activities:

“Before, I could not be assiduous and motivated.” (P11, INT1, and O)

“At [Name of fitness center #1], I did not really know what to do. So, I did not feel comfortable with the exercises.” (P8, INT1, and NO)

“I did not see any result, so there was discouragement.” (P10, INT1, and O)

Later in the program, some people mentioned that other barriers, such as financial means or pain, were added and diverted them from continuing in activities. Motivation and participation in activities declined in those whose constraints were heavy, making them vulnerable to dropout and reorienting their goals or commitment. This was more often the case in participants noncompleting the program:

“The difficulties were related to my health...It certainly would have helped if I was in better form when leaving. I had to give up after the 8^th^ training session because I was too tired after dialysis.” (P8, INT2, and NO)

“It was the financial aspect that stopped me. I would have liked to continue...I should have continued, but it became difficult...[Name of fitness center #2] was cheaper, except that I did not receive personal follow-up like here, so it did not interest me.” (P6, INT2, and NO)

Financial constraints have also been cited in other qualitative studies of adherence or perseverance [21, 22].

The 10 study participants were able to comment about the realities and barriers associated with changing their lifestyle. Many expressed that changing their lifestyle involved a considerable effort both for themselves and for their entourage. They knew what to expect, hoping that this time the experience would be easier and that changes would be visible in the short- and long-term. In the end, some people spoke about similar experiences and similar emotions:

“It is difficult to change…” and “It's easy to fall back into old habits…”

Another reality of cardiovascular disease prevention programs is the importance of setting realistic goals [23]. On average, participants had achieved one objective through the program, while it was found that they planned to attain at least 3 objectives. Despite finding that they had not achieved all their goals, participants were aware that they had progressed in the process of changing their lifestyle and were able to achieve positive results through negative experiences:

“Even if I did not reach all my goals, I think they were realistic...at first it was not obvious, but over time it became easier.” (P1, INT2, and O)

On the other hand, the low goal attainment rate among many participants may have decreased their “perception of personal efficacy” and their “motivation,” two factors that are discussed below. Study participants who completed the program or their commitment came out with a somewhat more positive perception of cardiovascular disease prevention programs, although the four who dropped out also saw positive results from their participation.

### 4.2. Theme No. 2: Perception of Self-Efficacy

#### 4.2.1. Influence of Previous Experiences on the Perception of Self-Efficacy

Individuals with better perception of control over their health status are more likely to participate in these prevention programs [24]. Indeed, from the interviews, it is suggested that the adoption of healthy lifestyles occurs naturally in some participants:

“I smoked 40 cigarettes a day. At age 44, I decided to stop completely...I threw away the remaining packs, put my lighter away and it was finished. It was as easy as that…I did not suffer and I do not regret it. I have always eaten well.” (P2, INT1, and NO)

“I changed a lot...I'm more aware. Sometimes it's easy to change things and sometimes it's difficult. At first, I found it hard to climb the stairs here, but in the long run it was easier. That is my personal motivation. I'm happy with myself. I did not think I would get there.” (P10, INT2, and O)

In contrast, it remains a difficult stage for others. Sometimes, it was even seen as an insurmountable challenge. Certain individuals take a negative stand to their behavior from the start, based on previous negative experiences:

“I've already tried to diet. It was far too complicated and it discouraged me. It was not at all realistic for me. I dropped out.” (P9, INT1, and O)

“I had already subscribed at another fitness center. We were not accompanied by a kinesiologist and were left to ourselves...I did not feel that I had worked hard, so there was some discouragement.” (P10, INT1, and O)

#### 4.2.2. Influence of Perception of Personal Efficacy on Quality of Life and Functional Capacity

There is evidence that a better perception of self-efficacy in individuals participating in a cardiac rehabilitation program increases, among other things, the functional ability and physical activity level [25]. This is also evident from the comments of our study participants. Those who were able to perceive concrete and positive changes in their quality of life had a better perception of personal efficacy later on. This confidence encouraged them to meet other challenges and achieve other positive results:

“I have less hypertension, less cholesterol, I feel more fit…there are reflexes that I did not have before.” (P10, INT2, and O)

“My perception is better, because I realized that at the cardiovascular level there is much improvement, less shortness of breath…I saw a lot of change since April with training…I see that it improves my condition of life. It lowers my cholesterol; it goes down more and more. I use my asthma inhaler less often.” (P10, INT2, and O)

Participants who completed the program showed a more confident and positive discourse than noncompleters. In the end, they expressed a better perception of their physical fitness and felt able to be physically active with greater ease. One can make connections with Bandura's theory, which confirms that behaviors can be predicted by the perception of personal efficacy [26].

#### 4.2.3. Perception of Personal Efficacy and Goals

It is noteworthy that, to avoid discouragement and demotivation and to increase the perception of personal efficacy, individualized objectives should be reviewed closely and periodically with kinesiologists [27]. While most participants expressed satisfaction with the program and found that it had helped them to progress along their journey, most did not succeed in meeting their objectives, and they felt that this could affect their perceived personal efficacy and discourage them at times:

“The training program required a lot of effort...I'm really not in shape, I get exhausted soon. I got bored ...You know...everybody exercises and you want to try and follow…then, it's not better. I spend the rest of the afternoon doing nothing because I'm exhausted. There is a lack of will on my part. It was very difficult for me to change my lifestyle...my goals, I did not achieve them.” (P8, INT2, and NO)

“It is very difficult for me to change lifestyle because of my family activities. We spend 6 evenings a week in the arena or at work…it is almost impossible to eat well. I still have incredible extra weight. I've been here for 6 months...without being discouraged, I'm looking forward to seeing a change in my weight.” (P9, INT2, and O)

On the other hand, we interpreted that for some participants, the perception of personal efficacy tended to vary during the program. Sometimes, for example, when they were very close to reaching a goal, they declared being more motivated and confident in achieving it, but at other times, they were less motivated:

“I have reached some of my goals...I must continue the program to reach other goals...One step at a time. I do not want to come up with too rigid requirements because I know myself. I have already done it and it has not given anything, so I want to be more realistic in my choices and assiduous through my decision...it is difficult to pass through these changes...but I am more aware of all this.” (P10, INT2, and O)

“…physical activity...lowered my stress a lot. I still have surplus weight but from the physical point of view and efficiency, it is 75 to 80% better. For now, I think it's already something. I come here twice a week, which was my initial goal and that's what I did to date. So, I tell myself that if I can do it for 6 months, I will be able to do it for another 6 months, perhaps integrating a better diet.” (P11, INT2, and O)

“It is easy for me to adopt good habits in life because I want to keep myself healthy. My motivation is totally between me and me...I'm always trying to reach my goals, but not all of them are achieved yet.” (P2, INT2, and O)

Some participants experienced difficulties during the program, and they nevertheless declared continuing to have a positive attitude towards their state of health. Here, a participant comments on the importance of setting realistic goals, which in his case may have enhanced his perception of personal efficacy:

“…You go from one goal to another without even realizing it, because your body begins to achieve better physical fitness and you find yourself in a situation where the goal you had set at the start seemed so difficult to reach and, so far, arrives easier than you would have believed. It is encouraging in this way to change some of our bad habits…sometimes you even say to yourself: this is not the goal that I should have set for myself.” (P3, INT2, and O)

Citations showed that those who had a greater perception of behavioral control and better “perception of personal efficacy,” even inconsistently during the program, were more satisfied with their journey. It was also noted that those who had a better perception of personal efficacy at the first interview were the most satisfied with their progress.

However, it can be seen that completers, and most noncompleters, conveyed difficulties that may have influenced their perception of personal efficacy, their degree of motivation, and their confidence in the future.

### 4.3. Theme No. 3: Motivation

Motivation is an important factor in cardiovascular disease prevention programs as it has been a recurrent theme in the pathway of all participants, completers, and noncompleters. Many realized, with the program, that they needed motivation to help them get through the process of lifestyle modification and not give up on the program:

“…One day I said to myself: I have to do something. I have a little 3-year-old boy, so I want to keep fit for when he grows up...I am motivated to train because of my son.” (P7, INT1, and NO)

For some, notably the less persevering participants with a low rate of attendance, motivation was difficult and contributed to dropout by 3 participants:

“There was a lack of will... I gave up.” (P8, INT2, and NO)

“Training is not something natural to me, so I have to motivate myself more than others.” (P11, INT2, and O)

#### 4.3.1. Influence of Goals on Motivation

The level of motivation was not constant among participants throughout the program. The initial level of motivation pointed out by most participants was very high. Motivation subsequently decreased in some, then increased at certain times. Several factors, such as the attitude and behavior of participants towards a specific objective, were found to be associated with motivation. One participant (P8) in particular, who was not at all confident in achieving his goals in the program from the start, was noticed by the kinesiologists. He abandoned the program after a few sessions. There was a sense of motivation manifested among those who were positive and enthusiastic about engaging in the process. Achievement of their goals as well as feedback and follow-up from kinesiologists provided during training sessions was felt to enhance the motivation of participants. On the other hand, failure to achieve a goal led to disappointments.

#### 4.3.2. Influence of Previous Experiences on Motivation

As mentioned earlier, previous experiences also influenced the motivation of participants. Since most had already undergone a training program, some feared that negative similar experiences could occur:

“…I was training at [Name of fitness center #2] and really did not like the experience. I dieted, I lost 60 pounds, regained them and then some. I was very discouraged and demotivated.” (P10, INT1, and O)

Other constraints (e.g., stress, lack of time, work, and health) were sometimes associated with a decline of motivation during the program. Participants who succeeded in finding a source of motivation (e.g., improving their condition) during difficult times expressed satisfaction with themselves and their progress and were those the most persevering in the program.

### 4.4. Theme No. 4: Feelings of Belonging in the Program and the Relationship of Trust

#### 4.4.1. Signing a Contract of Objectives

The use of “goal achievement contracts” conveyed many positive aspects for participants. Some mentioned that when they felt the urge to give up everything or when they were demotivated, the coach could easily remind them of the agreement made between them by showing them their signature below the target on the contract of success. The mere fact of having a contract in hand and signing was felt to allow everyone to develop “feelings of belonging in the program,” that it was “part of something” and that this something was a serious approach.

### 4.5. Supervision/Monitoring

Several participants commented on the fact that they needed to feel “guided.” “Setting realistic goals” with the contract and being under the supervision of a coach helped many to stay motivated and to not give up. Monitoring was, therefore, a priority for participants and they were aware of it from the start. Participants commented on the importance of follow-up by their kinesiologist to motivate and lead them with their supervision:

“I do not really agree with doing my own exercises, because you cannot control yourself...I'm not a specialist…it's important to have someone control what you do.” (P2, INT1, and O)

“What I like here is that there is someone with me. I have already been to a center and I am not interested in arriving on my own since no one tells me what to do. You do exercises and you think you do well. Here, at least you have someone to tell you what to do and correct you…I need that.” (P8, INT2, and NO)

“You always have someone watching you when you work, you get feedback right away.” (P1, INT2, and O)

Thus, participants' perceptions of the “relationship of trust” established with the kinesiologist were important factors in their behavior and in the success of their goals. Through feedback from kinesiologists and the second interview, this factor was explained by the fact that friendship was developed between the participant and the kinesiologist, and it became more difficult to “cheat” and to abandon the program. In addition, by looking at the many efforts made, participants who wanted to achieve their goals probably did not want to disappoint the person helping them to get on the path to success:

“Without the support of my kinesiologist, I would have done as usual, I would have let go, for sure. It helped me a lot to have someone with me. Even when I'm out there, he calls me the day before...that's what made me come here.” (P9, INT2, and O)

“…You are supervised by a person who has a lot of knowledge to guide you towards your goals.” (P3, INT2, and O)

It was seen that supervised coaching is a good way to increase adherence to training. For many participants, being part of a program in which they are involved, morally, physically, and financially has enabled them to realize that follow-up is a good remedy of demotivation:

“Sometimes it's hard to be motivated. What is stimulating here is that coaches [kinesiologists] motivate us a lot” (P10).

It has also been shown that in places where the center is more intimate and less busy, monitoring becomes easier to control, interactions are perhaps more frequent than in other places, so the atmosphere becomes ideal to develop bonds of friendship. The narrow link with an exercise specialist can prove to be a facilitator by fostering camaraderie [28, 29]:

“I find that a small clinic like this one, with a more personal touch, is more effective than big centers [Name of fitness center #1].” (P6, INT1, and NO)

### 4.6. Degree of Participation in Various Activities Related to the Program

Finally, the level of participation in various activities may have an impact on everyone's sense of belonging in the program. In this study, we found that those who participated at several levels in the program voiced a good understanding of cardiovascular disease prevention programs and were satisfied with their progress. In addition to indoor training, participants had access to health pamphlets and were encouraged to record their activities in a logbook. Individuals who participated in most activities and exercise sessions appeared to be more satisfied at the end of the study than those who were absent. On the other hand, those who left before the end of their contract or those who decided not to continue the program (*n* = 4) mentioned more barriers (financial, health conditions, lack of time, etc.) than other participants (Table 5).

## 5. Strengths and Limitations

Length of intervention was one of the strengths of our study, with an average duration of 6 months [6]. Also, we included not only participants who completed the program but also those who did not. Their individual testimonies were of the utmost importance in helping to understand dropout and its “mechanisms.” Results indicate that these 2 groups had different life experiences prior to entry in the program and different experiences in it. Therefore, an individualized approach is of utmost importance, as demonstrated by other researchers in an intervention of a similar duration [27]. Prior exercise experience (or lack thereof) conditions new experiences. A limitation of our study was that it was a convenience sample of volunteer participants who could afford enrollment in the program. Its composition was largely male with a homogenous ethnic background. Indeed, it was shown that gender might be a factor influencing cardiac rehabilitation [30]. Finally, the generalization of the findings may be limited to contexts similar to our case study.

## 6. Conclusion

Some common discourses were shared by participants. One in particular was that they were, with different degrees, made aware to cardiovascular disease risk factors and were able to identify them. People who enroll in such a program are already a minority, as reported in the literature [31]. A favorable attitude must be present to take a step towards entry in the program. Already during the first interview, the information they provided indicated how there were differences between those who completed 6 months versus those who did not. However, a big difference was in the way they dealt with barriers: noncompleters expressed more difficulties in finding solutions to barriers. It is therefore important to plan some support for individuals facing difficulties to continue the exercise program.

Self-efficacy seemed to be at the center of the process. It was generally stronger in completers, and perception of self-efficacy varied according to difficulties in reaching goals that may have been influenced by other than individual factors. Self-efficacy should be addressed from the beginning of enrollment in such programs. Indeed, the history of both completers and noncompleters revealed that their perception of self-efficacy was strongly influenced by their past physical activity experience. Indeed, a meta-analysis of qualitative studies in cardiac rehabilitation, Neubeck et al. [14] identified the perception of lack of self-efficacy over risk factors as a barrier. Our participants also mentioned this barrier.

Lack of motivation, monetary reasons, and failure to achieve their set goals were the most common reasons for noncompletion or dropout. Financial problems are found to be quantitative as much as qualitative as a real barrier in countries where cardiac rehabilitation is not covered by insurance or the public health system. Even as a promising approach, cardiac rehabilitation has a major accessibility problem, at least in North America [5, 32], which is likely to deter perseverance in those who cannot cover costs.

Close supervision by kinesiologists was a characteristic that all participants perceived as being very positive and helpful in their progress as well as a way to increase their confidence and motivation. The signing of a contract and other materials (logbook, etc.), supervision, and follow-up helped to create a sense of belonging, favoring the completion of participation by increasing motivation. However, such programs must also consider ways to empower participants since they will have to integrate their new lifestyle into their own constraints [33].

## Figures and Tables

**Table 1:** Information on the characteristics of participants. CVD: cardiovascular disease.

Nonmodifiable CVD risk factors	Participant #										Total
	P1	P2	P3	P4	P6	P7	P8	P9	P10	P11	
(i) Heredity		x		x	x			x	x	x	6/10
(ii) Age (men ≥50 years, women ≥60 years)		x	x		x						3/10
(iii) Being male	x	x	x	x	x	x	x	x			8/10
Modifiable CVD risk factors	P1	P2	P3	P4	P6	P7	P8	P9	P10	P11	Total
(i) Smoking					x	x					2/10
(ii) High blood pressure	x		x		x		x	x	x	x	7/10
(iii) Dyslipidemia	x			x			x		x		4/10
(iv) Overweight^∗^	x	x	x	x	x		x	x	x	x	9/10
(v) Sedentary behavior	x	x	x	x	x	x	x	x	x	x	10/10
(vi) Diet rich in trans and saturated fats, sugar, and sodium	x		x	x	x		x	x	x	x	8/10
(vii) Stress	x		x	x	x			x	x		6/10
Other health problems		x	x		x	x	x	x	x	x	8/10
(i) Diabetes			x								1/10
(ii) Myocardial infarctus			x		x						2/10
(iii) Atherosclerosis			x		x					x	3/10
(iv) Coronary artery bypass surgery			x		x						2/10
(v) Kidney problems							x				1/10
(vi) Crohn's disease						x					1/10
(vii) Myocarditis, viral		x									1/10
(viii) Arrhythmia		x								x	2/10
(ix) Gout problem								x			1/10
(x) Arthritis		x							x		2/10
(xi) Asthma									x		1/10
(xii) Has a job	x		x	x			x	x		x	6/10
(xiii) Work termination (health reasons)					x	x					2/10
(xiv) Retired		x							x		2/10
(xv) Married/common-law partner	x	x		x	x	x	x	x	x	x	9/10
(xvi) Separated/divorced/other			x								1/10
(xvii) Medications	x	x	x	x	x	x	x	x	x	x	10/10
(xviii) Body mass index	28.3	26.5	29.7	36.0	26.4	19.7	38.4	37.7	32.8	26.3	
(xix) Waist circumference (cm)	100	104	106	116	106.5	86	121	115	114	93	
(xx) Fat (%)	25.9	28.7	31.6	30.6	29.7	13.7	39.7	25.7	38.7	39.9	

P5: did not return our call for the follow-up interview and was not included in the analysis.

**Table 2:** Reasons for participation in the program.

Reasons	Participant #										Total
	P1	P2	P3	P4	P6	P7	P8	P9	P10	P11	
(i) To feel better		x	x	x	x	x	x			x	7/10
(ii) Look better								x			1/10
(iii) Have more energy	x			x		x	x		x	x	6/10
(iv) Sleep better		x								x	2/10
(v) Control weight	x	x	x		x		x	x	x		7/10
(vi) Better health	x	x	x	x	x			x	x		7/10
(vii) Have fun						x					1/10
(viii) Control stress		x						x			2/10
(viii) More self-confidence		x									1/10
(ix) Decrease boredom/meet new people		x									1/10
(x) Feel stronger											0/10
(xi) Other reasons								x	x		2/10

**Table 3:** Reasons for participants' sedentary behavior.

Reasons	Participant #										Total
	P1	P2	P3	P4	P6	P7	P8	P9	P10	P11	
(i) Lack of time	x		x	x			x	x	x	x	7/10
(ii) Lack of interest			x				x	x		x	4/10
(iii) Lack of knowledge											0/10
(iv) Age					x						1/10
(v) Too difficult					x		x				2/10
(vi) Fear of getting hurt						x					1/10
(vii) Fear of heart attack					x			x			2/10
(viii) Too expensive										x	1/10
(ix) Negative influence from entourage											0/10
(x) Health does not allow it			x			x					2/10
(xi) Difficult access to center											0/10
(xii) Transportation problems											0/10
(xiii) No access to a competent kinesiologist								x		x	2/10
(xiv) Lack of motivation											0/10
(xv) Other											0/10

**Table 4:** Four themes that may influence participation and adherence to a primary and secondary CVD prevention program.

Themes	Subthemes
No. 1 knowledge and awareness of the disease	Risk factor awareness
	Identifying causes of the problem versus understanding causes of the problem
	Participants' knowledge of risk factors
	Learning to recognize and identify barriers and realities related to physical activity practice

No. 2 perception of personal efficacy	Influence of previous experiences on the perception of personal efficacy
	Influence of perception of personal efficacy on quality of life and functional capacity
	Influence of perception of personal efficacy on objectives

No. 3 motivation	Influence of objectives on motivation
	Influence of perception of personal efficacy on motivation
	Influence of previous experiences on motivation

No. 4 feelings of belonging in the program and the relationship of trust	Signing an objectives contract
	Supervision/follow-up
	Degree of participation in various activities related to the program, e.g., logbook, health capsules and training
	Influence on motivation

**Table 5:** Comparison of 4 factors that may influence participation and adherence to primary and secondary CVD prevention programs for completing and noncompleting participants.

Theme	Completers (*n* = 4)	Noncompleters or dropouts (*n* = 6)
Knowledge or awareness of the disease	Made aware to risk factors + after participating in the program	
	Detect alarm signals before onset of the disease	Detect alarm signals, often after onset of the disease
	Identify risk factors related to their health problem at start and finish	
	Target: preventing the disease	Target: treating the disease
	Seem to have acquired more knowledge about risk factors, especially in those completing the program	Seem to have acquired risk factor knowledge. Aware of their condition but willingness to make changes
	Capable of identifying barriers to physical activity and finding solutions, especially after completion of the program	Capable of identifying barriers to physical activity, but have more difficulty in countering them (illness, cost, etc.)
	Have a better ability to self-identify in a context of training or lifestyle changes in the end	

Perception of personal efficacy	Those who have better perception of personal efficacy at the outset are more satisfied with their journey	
	Previous experiences influence their perception of personal efficacy (+ and -)	
	+ Confident and positive about achieving their goals during the program	A little - confident and positive to achieve their goals during the program
	Better perception of their physical fitness at the end of the program	In general, - better perception of their physical fitness than completers
	Feel able to perform healthy behaviors with greater ease through the program	With difficulties and barriers experienced through the program, feel - able to perform behaviors with ease
	Perception of personal efficacy varies, often depending on the proximity of reaching a goal	
	Difficulty in achieving goals, affecting self-efficacy and causing discouragement	

Motivation	Essential to the process of lifestyle change in all study participants	
	Motivation varies through the program: achievement of objectives, feedback and follow-up by kinesiologists enhance their motivation	
	Lack of motivation: significant barrier to adherence to physical activity	Lack of motivation: barrier that causes participants to give up
	Previous experiences influence their motivation (initially and throughout the program)	
	Motivation + great among those who are initially positive to engage in this process and willing to pursue it	Less enthusiastic than persevering participants to continue in this step in the end

Feeling of belonging in the program and the relationship of trust	Signing a contract for achieving goals develops “a sense of belonging in the program”	
	Recognize that tracking from their kinesiologists to motivate and lead them in their coaching is an essential part of these programs	
	“Relationship of trust” established with kinesiologists was an important factor in participants' behavior and the success of their objectives.	“Relationship of trust” established with kinesiologists was not so well developed due to lack of adherence to activities
	Increased sense of belonging in the program by participating in most activities (health capsules, exercise sessions, etc.)	Have less developed a sense of belonging in the program by abstaining from many activities (health capsules, exercise sessions, etc.)

## Data Availability

The qualitative data used to support the findings of this study are restricted by the Comité d'éthique de la recherche avec des êtres humains (Ethics Review Board in Human Participants) in order to protect patient privacy. Data could be available following an informed consent of each participant from researchers. As stated by Chauvette et al. 2019: ‘There is a unique engagement between participant and researcher in generating the data, which is often rich and contextual. However, despite the growing movement toward providing open access to data precipitated by requirements of some funding bodies, it is not appropriate to share some qualitative data from transcripts, field, or reflective notes. This position is supported by epistemological, methodological, legal, and ethical principles.' Chauvette, A. et al. (2019). Open Data in Qualitative Research. International Journal of Qualitative Methods, 18, 1609406918823863.
